# Genome Haploidisation with Chromosome 7 Retention in Oncocytic Follicular Thyroid Carcinoma

**DOI:** 10.1371/journal.pone.0038287

**Published:** 2012-06-01

**Authors:** Willem E. Corver, Dina Ruano, Karin Weijers, Wietske C. E. den Hartog, Merlijn P. van Nieuwenhuizen, Noel de Miranda, Ronald van Eijk, Anneke Middeldorp, Ekaterina S. Jordanova, Jan Oosting, Ellen Kapiteijn, Guido Hovens, Jan Smit, Tom van Wezel, Hans Morreau

**Affiliations:** 1 Department of Pathology, Leiden University Medical Center, Leiden, The Netherlands; 2 Department of Clinical Oncology, Leiden University Medical Center, Leiden, The Netherlands; 3 Department of Endocrinology, Leiden University Medical Center, Leiden, The Netherlands; Cardiff University, United Kingdom

## Abstract

**Background:**

Recurrent non-medullary thyroid carcinoma (NMTC) is a rare disease. We initially characterized 27 recurrent NMTC: 13 papillary thyroid cancers (PTC), 10 oncocytic follicular carcinomas (FTC-OV), and 4 non-oncocytic follicular carcinomas (FTC). A validation cohort composed of benign and malignant (both recurrent and non-recurrent) thyroid tumours was subsequently analysed (n = 20).

**Methods:**

Data from genome-wide SNP arrays and flow cytometry were combined to determine the chromosomal dosage (allelic state) in these tumours, including mutation analysis of components of PIK3CA/AKT and MAPK pathways.

**Results:**

All FTC-OVs showed a very distinct pattern of genomic alterations. Ten out of 10 FTC-OV cases showed near-haploidisation with or without subsequent genome endoreduplication. Near-haploidisation was seen in 5/10 as extensive chromosome-wide monosomy (allelic state [A]) with near-haploid DNA indices and retention of especially chromosome 7 (seen as a heterozygous allelic state [AB]). In the remaining 5/10 chromosomal allelic states AA with near diploid DNA indices were seen with allelic state AABB of chromosome 7, suggesting endoreduplication after preceding haploidisation. The latter was supported by the presence of both near-haploid and endoreduplicated tumour fractions in some of the cases. Results were confirmed using FISH analysis. Relatively to FTC-OV limited numbers of genomic alterations were identified in other types of recurrent NMTC studied, except for chromosome 22q which showed alterations in 6 of 13 PTCs. Only two *HRAS*, but no mutations of *EGFR* or *BRAF* were found in FTC-OV. The validation cohort showed two additional tumours with the distinct pattern of genomic alterations (both with oncocytic features and recurrent).

**Conclusions:**

We demonstrate that recurrent FTC-OV is frequently characterised by genome-wide DNA haploidisation, heterozygous retention of chromosome 7, and endoreduplication of a near-haploid genome. Whether normal gene dosage on especially chromosome 7 (containing *EGFR*, *BRAF*, *cMET*) is crucial for FTC-OV tumour survival is an important topic for future research.

**Microarrays:**

Data are made available at GEO (GSE31828).

## Introduction

Thyroid cancer is a common disease of the endocrine system with a worldwide incidence rate of approximately 212,000 new cases and 35,000 related cancer deaths per year [1]. Differentiated thyroid cancer can be subdivided into medullary (MTC) and non-medullary (NMTC) thyroid carcinoma. Differentiated NMTC is the most common, and two subgroups, papillary thyroid cancer (PTC) and follicular thyroid cancer (FTC), account for approximately 95% of all thyroid cancers. Many variants of these subgroups have been identified, including a follicular variant of PTC (FVPTC) and the Hürthle cell or oncocytic variant of FTC (FTC-OV).

The large majority of patients with differentiated thyroid carcinoma have a favourable prognosis and patient cure is achieved by thyroidectomy, followed by adjuvant radioactive iodine treatment. However, approximately 5% of patients show recurrent disease, mostly due to impaired radioactive iodine-response, frequently leading to death within 5 years of surgical intervention [2]. Treatment options for these recurrent cases remain limited.

Oncocytic carcinomas are characterised by mitochondrial hyperplasia, which gives rise to distinctive morphologic features typified by a strong eosinophilic cytoplasm in conventional histology. The accumulation of mitochondria is associated with mutations of mitochondrial DNA (mtDNA) in one of the three energy-transducing enzyme complexes of the respiratory chain. In the FTC-OV, mtDNA mutations are mostly found in NADH-ubiquinone oxidoreductase of complex I [3], resulting in loss of enzyme activity. Due to the dysfunction of oxidative phosphorylation, cells become dependent on glycolysis for energy production.

Progress has been made in further understanding the underlying genetic alterations in thyroid cancer. In NMTC chromosomal aberrations encompassing chromosomal losses or gains have been described [4]–[6] although no specific pattern was recognized (recently reviewed in [7] and [8]). One study suggested a relation between numerical chromosomal aberrations, oncocytic follicular thyroid carcinoma and recurrence [9]. *BRAF* and RAS mutations, and *RET*-chromosomal rearrangements (*RET/PTC* and *RET/NTRK1*) have been identified in approximately 35–70% of cases of PTCs [10]. The *BRAF* V600E mutation was also found in 26% of FVPTC cases [11]. Interestingly, *BRAF* and RAS mutations, and *RET/PTC* rearrangements appear to be mutually exclusive [6], [12], and the less common *RET/NTRK1* rearrangement, present in 5.5% of the PTCs, is absent from *RET/PTC*-associated PTC [13]. While *BRAF*-RAS mutations and *RET*-chromosomal rearrangements are rarely seen in FTCs [14], *PAX8/PPARγ* rearrangements are seen at frequencies of 30–40% [15] and *PIK3CA* copy number gains and mutations have also been recently found in FTCs [16]. While all of these somatic DNA alterations are known to be involved in the PIK3CA/AKT and MAPK pathways, the upstream receptors of these signalling pathways, *EGFR* and *VEGFR1*, also display copy number gains in about 32% and 44% of FTCs, respectively [16]. These studies strongly implicate the PIK3CA/AKT and MAPK signalling pathways in thyroid carcinogenesis and suggest that tailored compounds targeting these pathways might be therapeutically beneficial. Phase II trials including a variety of multi-kinase inhibitors are ongoing or have been completed (reviewed by Kapiteijn et al. [17]) and partial responses and stable disease were observed in patients with differentiated thyroid carcinoma [18].

Many of the abovementioned studies used primary NMTC tissue, without further information on recurrence in the patient. In order to better characterise recurrent NMTC, we analysed primary tumour tissue from a cohort of twenty-seven NMTC patients showing recurrence, comprising mainly PTCs or FTC-OV tumours. These patients were previously enrolled in a clinical trial with sorafenib, a multi-kinase inhibitor targeting the MAPK pathway [17], [19]. We carried out a DNA copy number analysis of tumour tissue using genome-wide SNP arrays and integrated information on DNA content into these data. By combining these data with allele-specific intensity, we were able to derive an estimation of the true chromosomal dosage in these tumours. We validated our findings in a cohort of twenty frozen benign and malignant (both recurrent and non-recurrent) thyroid tumours. This analysis was complemented by mutation analysis of molecular components of the PIK3CA/AKT and MAPK pathways.

## Materials and Methods

### Patient material and flow cytometry

Twenty-seven patients with recurrent non-medullary thyroid carcinoma were included in the study. These patients were enrolled under an earlier study protocol [19], approved by the Institutional Review Board of the Leiden University Medical Center. This study has been registered at ClinicalTrials.gov (# NCT00887107). Informed written consent was obtained from all patients in the study.

Formalin fixed paraffin embedded (FFPE) primary thyroid carcinoma samples of the primary study were obtained from a number of pathology departments in the Netherlands. For validation frozen primary thyroid tumour samples were selected from twenty patients (Dept. of Pathology, LUMC). One of these patients took also part in the primary study (primary study Table 1, No. 9; validation study Table 2, No. 39).

**Table 1:** Summary of results of the analysis of 27 recurrent NMTC tumours: DNA index, allelic state and chromosome copy number determined by FISH for chromosomes 6 (centromeric probe) and 7 (centromeric and EGFR locus specific probes) and mutation analysis of *BRAF*, *EGFR*, RAS (*HRAS, KRAS* and *NRAS*) genes and *PIK3CA*.

Patient No.	Tumour type	Gender	Age at diagnosis	DNA index#	Chr. 6 allelic state (centromeric region)	Chr. 6 FISH copy number	Chr. 7 allelic state (centromeric region)	Chr. 7 FISH copy number	*BRAF*	*EGFR*	RAS	*PIK3CA*
1	FTC	m	74	1.01	AB	nd	AB	nd	−	−	*NRAS*	−
2	FTC	f	59	0.98	AB	nd	AB	nd	−	−	−	−
3	FTC	m	82	0.90	AB	nd	AB	nd	−	−	−	−
4	FTC/FVPC	f	65	1.02	AB	nd	AB	nd	−	−	−	−
5	FTC-OV	m	77	0.73	AAAA	2	AB	2	−	−	−	−
6	FTC-OV	m	68	0.71	A	1	AB	2	−	−	−	−
7	FTC-OV	m	59	0.67	A	1	AB	2	−	−	−	−
8	FTC-OV	m	54	1.27	AA	2	AABB	4	−	−	−	−
9	FTC-OV	m	60	1.19	AA	2	AABB	4	−	−	−	−
10	FTC-OV	m	69	1.01	A	1	AABB	4	−	−	−	−
11	FTC-OV	f	66	1.28	AA	2	AAB	3–4 *	−	−	**−**	**+**
12	FTC-OV	f	61	**0.62**/1.26	A	1	AB	4	−	−	−	−
13	FTC-OV	f	59	**0.53**/1.04	A	nd	AB	nd	−	−	−	−
14	FTC-OV	f	53	0.98/**1.22**	AA	2	AABB	4	−	−	−	−
15	FVPC	m	68	1.05	AB	nd	AAB	nd	−	−	−	−
16	PTC	m	64	1.00	AB	nd	AB	nd	**+**	**−**	−	−
17	PTC	f	65	1.02	AB	2	AB	2	**+**	**−**	−	−
18	PTC	m	62	2.06	AABB	nd	AABB	nd	**+**	**−**	−	−
19	PTC	m	72	**1.02**/2.05	AB	2	AB	2	**+**	**−**	−	−
20	PTC	m	62	1.07	AB	nd	AB	nd	**+**	**−**	−	−
21	PTC	f	60	0.92	AB	2	AB	2	−	−	−	−
22	PTC	f	77	**0.98**/2.02	AB	nd	AB	nd	−	−	*NRAS*	−
23	PTC	m	60	1.01	AB	nd	AB	nd	**+**	**−**	−	−
24	PTC-OV	f	72	0.94	AB	nd	AB	nd	**+**	**−**	−	−
25	PTC-OV	m	56	0.97	AB	nd	AB	nd	**+**	**−**	−	−
26	PTC-OV	f	63	1.04	AB	nd	AB	nd	**+**	**−**	−	−
27	PTC Tall Cell	m	79	1.00	AB	nd	AB	nd	**+**	**−**	−	**+**

Underlined: discrepancy between FISH copy number and allelic state.

# = bold indicates the dominant population as determined by flow cytometry, in cases where multiple populations are present.

* = an intermingled pattern of three and four copies was found both for the centromeric and the EGFR locus specific probes.

+ = presence of mutation.

− = mutation not found.

nd = not determined.

**Table 2:** Summary of results of the analysis of the 20 tumours in the validation cohort: diagnosis, near-homozygous phenotype and recurrence.

No.	Diagnosis	Near-homozygous phenotype	Recurrence
28	FA	No	-
29	FA	No	-
30	FA, partially OV	No	No
31	ATC	No	*
32	ATC	No	*
33	ATC	No	*
34	ATC	No	*
35	ATC, partially OV	No	*
36	FTC	No	**Yes**
37	FTC, partially OV	**Yes**	**Yes**
38	FTC-OV&	**Yes**	*
39	FTC-OV**	**Yes**	**Yes**
40	FTC-OV, min. inv.	No	No
41	FTC-OV, min. inv.	No$	No
42	Mixed FTC/PTC	No	**Yes**
43	PTC	No	No
44	PTC	No	No
45	PTC	No	No
46	PTC	No	No
47	PTC	No	**Yes**

The presence of homozygous chromosomes was determined by high-density SNP-arrays.

min. inv. = minimal invasive.

* = incomplete resection and death of disease <5 months.

** = sorafenib study, FFPE sample No. 9.

& = other tumour fraction dedifferentiated to ATC.

$ = possible intratumour heterogeneity.

Samples were handled according to the medical ethical guidelines described in the Code Proper Secondary Use of Human Tissue established by the Dutch Federation of Medical Sciences (www.federa.org). Paraffin or frozen sections were taken from all samples, haematoxylin and eosin stained, and reviewed by a pathologist (HM).

### Multiparameter DNA flow cytometry of FFPE sample

Cell suspensions were prepared for multiparameter DNA flow cytometry from three to four 2 mm diameter FFPE tissue punches (Beecher Instruments, distributed by K7 Biosystems Inc. Chicago, IL). Punches were dewaxed and submerged in citrate buffer for heat-induced antigen retrieval (HIAR) at 80°C for 60 min, followed by dissociation using collagenase/dispase at 37°C. Harvested cells were washed, counted and stored on ice. Next, cell suspensions were indirectly labelled for stromal cells (allophycocyanin, APC), epithelial cells (fluorescein isothiocyanate, FITC) and DNA (propidium iodide, PI) simultaneously and analysed on an LSRII flow cytometer (BD Biosciences, Erembodegem, Belgium) [20].

### DNA Isolation

In order to enrich for tumour DNA, 0.6 mm diameter tissue punches (Beecher Instruments) were taken from selected tumour areas. From the frozen samples three to six 10 µm sections were taken using a cryostat. DNA from FFPE samples or frozen samples was isolated by an overnight or 2 hour digestion with proteinase K at 56°C, respectively. Next (morning), DNA was purified using the NucleoSpin purification kit (Macherey-Nagel GmbH & Co. KG, Düren, BRD) according to the manufacturer's instructions. DNA concentrations were determined using the Picogreen method (Life Technologies Europe BV, Bleiswijk, The Netherlands).

### SNP Array Analysis

In the initial series 6k SNP arrays (GoldenGate assay, Illumina, Eindhoven, The Netherlands) experiments were performed at the Leiden Genome Technology Center (http://www.lgtc.nl), as described with minor modifications: [21] 0.4–1.0 µg DNA was used as input in a multi-use activation step and was subsequently dissolved in 60 µl resuspension buffer. Genotype, genotype quality call score (GCS), and the allele specific raw intensities were extracted using Beadstudio 2.3 (Illumina,). The beadarraySNP package was adapted to combine copy number profiles, allele-specific intensities and the DNA index, allowing the determination of the allelic state for all genomic regions [22].

Briefly, the normalised intensity signal (copy number) and the so-called lesser allele intensity ration (LAIR) values are subjected to a segmentation procedure, which allows the identification of genomic regions that share the same allelic state [21]. The LAIR value is a measure of the contribution of the two parental alleles to a genomic region. This value is close to 1 when the contribution of both alleles of a SNP to the total intensity in the tumour is similar to that of the reference sample. The value is close to 0 when there is no signal for one of the alleles in the tumour (as in LOH). Allelic imbalances will show intermediate values. Regions with a lesser allele intensity ratio close to 1 should have an even copy number because both alleles are in balance. The calculated DNA index obtained by LAIR analysis (LAIR index) from the summing of the number of alleles for each SNP should match the DNA index measured by flow cytometry. This is used to calibrate the allelic copy number in each genomic region, referred to as the “allelic state”. The following allelic states can be distinguished: [AB], normal; [A], LOH or physical loss in the context of a diploid genome but monosomy in the context of a haploid genome; [AA], copy-neutral LOH; [AAA] or [AAAA] etc., amplified LOH; [AABB] or [AAABBB] etc., amplified heterozygous state; [AAB], [AAAB], [AAABB] or [AAAABB] etc., imbalanced gain. All array data is MIAME compliant and the raw data has been deposited in GEO (http://www.ncbi.nlm.nih.gov/geo/query/acc.cgi?token=fnibdumqmooeezu&acc=GSE31828).

For the validation series CytoSNP-12 (Illumina, USA) high-density SNP-arrays were used according to the manufacturer instructions. After HE guided evaluation of snap frozen tumours the tissue was sectioned. DNA was isolated as described above and 4 µl of DNA was processed according to the Infinium HD Ultra Assay protocol and used for hybridization. Sample processing and hybridisation was performed by ServiceXS (Leiden, The Netherlands).

### Interphase FISH analysis

The chromosome 7 centromeric region probe (p7t1 alphoid satellite probe) and the α-satellite centromeric chromosome 6-probe (D6Z1, Oncor, Gaithersburg, MD) were labelled with biotin by standard nick-translation. For *EGFR*, PAC clone RP5-1091E12 was selected from the Ensembl Genome Browser and was labelled with digoxigenin-12-dUTP (Roche, Basel, Switzerland) by standard nick translation. The hybridisation solution contained 50% formamide, 10% dextran sulphate, 50 mM sodium phosphate (pH = 7.0), 2× SCC, 3 ng/µl of each probe, and a 50-fold excess of human Cot-1 DNA (BRL-Life Technologies, Rockville, MD, USA).

For interphase FISH 500 to 2000 nuclei were prepared from nine FTC-OV and three PTCs, spotted on ethanol-cleaned and air-dried glass slides. After spotting, the slides were dried overnight at room temperature. If necessary, the slides were incubated in a 0.1 M solution of Na_2_B_4_O_7_ to permit swelling of the nuclei. The slides were then serially rinsed in PBS and sterile water, dehydrated and air-dried before hybridisation. After applying the probe mix the slides were heated at 80°C for 12 min and incubated overnight in a moist chamber at 37°C. Next, the slides were further prepared for immunodetection, and fluorescent spots of a minimum of 50 nuclei were counted per probe combination. Images were captured using a COHU 4910 series monochrome CCD camera (COHU, San Diego, CA, USA) attached to a DM fluorescence microscope (Leica, Wetzlar, Germany), equipped with a PL Fluotar 100×, NA 1.30 – 0.60 objective and I3 and N2.1 filters (Leica) and Leica QFISH software (Leica Imaging Systems, Cambridge, UK).

### Somatic mutation analysis

For somatic mutation analysis of *EGFR* (exons 18–21), the RAS (*HRAS*, *KRAS* and *NRAS*) genes (codons 12, 13 and 61), *PIK3CA* (codons 542, 545 and 1047) and *BRAF* (codon 600), Sanger sequencing was performed using specific primers listed in Table S1. To allow universal sequencing, M13 tails were added to all primers, which were obtained from Eurofins (Ebersberg, Germany).

Uniform PCR conditions were used (iCycler, Bio-Rad, Veenendaal, The Netherlands) in 10 µl reactions with 10 ng DNA, iQ Supermix (Bio-Rad) and 2 pmol primers, as described. PCR conditions were: 10 minutes at 95°C, followed by 40 cycles of 5 seconds at 95°C, 10 seconds at 60°C, and 10 seconds at 72°C, with a final elongation step of 10 minutes at 72°C. Purified PCR products were Sanger sequenced at the Leiden Genome Technology Center and analyzed using the Mutation Surveyor software package (Softgenetics, PA, USA).

## Results

### Patient cohort

We analyzed twenty-seven recurrent non-medullary thyroid carcinoma (NMTC) cases. Tumours were categorised according to their histological subtype. The FTC-OV and PTC variants were predominant in this series (for details, see Table 1). To validate our findings we further analyzed a cohort of 20 patients, primarily composed of ATC, FTC-OV and PTC (Table 2 and Table S2).

### SNP-array analysis, multiparameter DNA flow cytometry and FISH

SNP array analysis of all ten FTV-OV showed genome-wide LOH on most of the chromosomes. In all cases heterozygosity was retained for chromosome 7. After integration of the DNA index (DI, see Table 1) in the SNP-array analysis 5/10 FTC-OV showed LOH due to chromosomal monosomy with the allelic state [A] (see the materials and methods). In the remaining 5 FTC-OV DI range (0.98–1.27) copy neutral LOH was found (allelic states [AA]). The latter suggests endoreduplication of a previous near-haploid genome.

**Figure 1:**
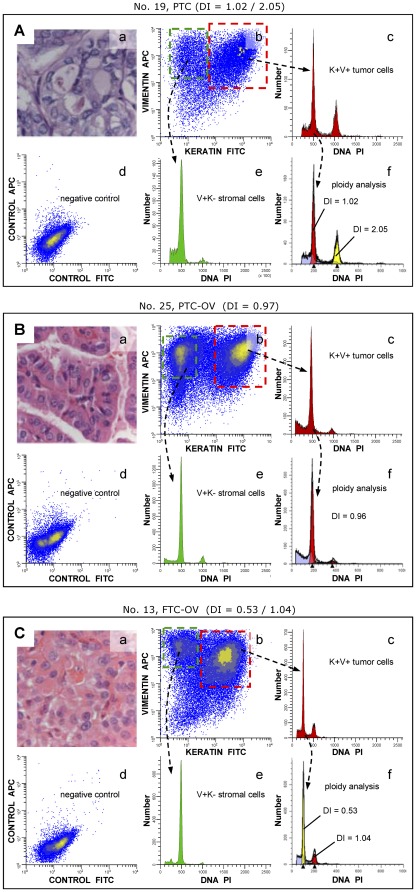
Examples of DNA content analysis of recurrent NMTC. Multiparameter DNA content analysis was performed on FFPE NMTC, as described. **A**. Multiparameter DNA content analysis of a bimodal PTC with a DI of 1.02 and 2.05 (case No. 19), **B**. a PTC-OV with a DI of 0.97 (case No. 25) and **C**. a bi-modal FTC-OV with a DI of 0.53 and 1.04, respectively (case No. 13). **a**. Haematoxylin – eosin staining 200×. **b**. keratin vs. vimentin density plot (note the vimentin co-expression of these tumours and the clear separation between the stromal and the epithelial cell fraction. The expression of keratin and vimentin are high, relative to the controls showing background fluorescence [**d**]). Twenty-five samples, 93% (25/27), showed high vimentin co-expression in more than 50% of the cancer cells (data not shown). **c**. DNA histogram generated after gating on the epithelial cell fraction. **e**. DNA histogram generated after gating on the normal DNA diploid stromal cell fraction. This fraction was used as a DNA content reference. **f**. DNA histogram of the epithelial cell fraction after modelling by ModFit (note that the presence of a second cell cycling population in the bimodal PTC and the FTC-OV DNA histograms is significant and demonstrates endoreduplication. In addition, the FTC-OV shows a dominant DNA near-haploid population [**c**, **f**]).

A single tumour population was observed in 81% of the samples, after gating in the flow cytometric analysis on the keratin-positive (K+) epithelial cell fractions (Figure 1). Remarkably, two out five FTC-OVs with a DNA near-haploid DI (range 0.53–0.73) showed a second cell population with a DI twice that of the DNA near-haploid population, indicative of endoreduplication of the DNA near-haploid population (Table 1, Figure 1).

**Figure 2:**
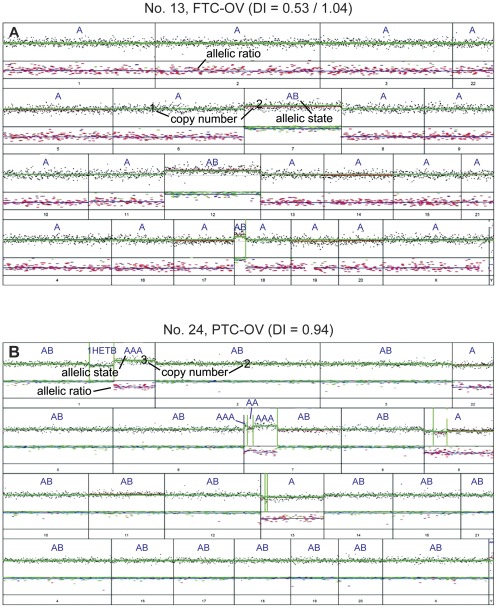
Examples of genome-wide allelic state analysis of an FTC-OV and a PTC-OV. **A**. FTC-OV (case No. 13, see also Figure 1) with DIs of 0.53 and 1.04 shows allelic state [A] for most chromosomes, except for chromosomes 7 and 12 and a segment of chromosome 18 showing retention (allelic state [AB]). Chromosome X also shows an allelic state [A]. **B**. The PTC-OV sample with a DI of 0.94 (case No. 24) shows a relatively limited number of genomic alterations. Chromosomes 1q and 7p showed an [AAA] allelic state after LAIR analysis. Another segment of 1q showed 1 copy but was heterozygous, which can be explained by a balanced mixture of two populations, one with an allelic state [A] and one with an allelic state [B], representing intra-tumour heterogeneity. In comparison with normal cells, one copy of chromosome 9, 13 and 22 was lost, as shown by the allelic state [A]. Both X chromosomes were detected in this female patient [AB].

**Figure 3:**
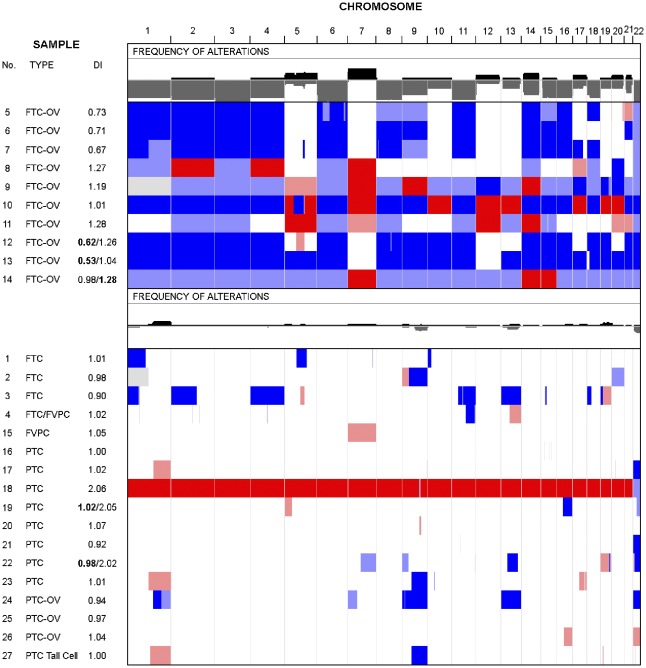
Summary of the genomic alterations found after LAIR analysis (see Materials and Methods) in 27 recurrent NMTCs. In this heatmap, rows represent tumours and columns represent chromosomes. The first column shows the tumour number, type and DNA index (DI). The tumours have been grouped according to their subtype, with ten FTC-OV tumours in the upper group and 17 NON FTC-OV in the lower group. The combined frequency of genomic alterations for each group is indicated in a separate row. Black indicates allelic states with >2 copies and at least one B allele retained, e.g. [AABB] and [AAB]. Grey indicates allelic states of [A] and [AA]. The colours in the heatmap indicate: white, allelic state [AB] = normal heterozygous state. Dark red, allelic states [AABB], [AAABBB], etc. = amplified heterozygous states. Light red, allelic states [AAB], [AAABB], etc. = imbalanced gain. Dark blue, allelic state [A] = LOH or physical loss in the context of a diploid genome but monosomy in the context of a haploid genome. Light blue, allelic states [AA], [AAA], etc. = copy neutral LOH and amplified LOH, respectively. Notice the retention of chromosome 7 for five out of ten FTC-OV tumours (allelic states [AB]) with the remaining five showing endoreduplication with allelic states [AABB] (n = 4) or [AAB] (n = 1). The Integrative Genomics Viewer (IGV) was used to produce this image.

An example of complete allelic state of a FTC-OV (case No. 13) in the SNP array analysis is shown in Figure 2A, whereas in Figure 3 all samples are depicted. The FTC-OV case No. 13 is only heterozygous [AB] for chromosomes 7, 12 and a segment of 18. All other autosomes show monosomy [A]. Flow cytometric analysis showed in FTC-OV cases with allelic states AA a DI between 1.01 and 1.28, indeed approximately twice the range of DIs found in the DNA near-haploid tumour fractions. One of these near-diploid tumours was also bimodal, with cell populations displaying DIs of 0.98 and 1.22, possibly reflecting intra-tumour heterogeneity. The near-haploidisation process, with or without subsequent endoreduplication in FTC-OV, implied different combinations of chromosomes, however LOH was seen in all cases for chromosomes 3, 6, and 22. Remarkably, this histological variant never displayed allelic states [A] or [AA] for chromosome 7 as all the FTC-OV samples showed retention of chromosome 7 in a heterozygous state (at least one copy of the B allele was preserved).

All other NMTC variants, with the exception of one PTC with a DI of 2.06 (Tumour No. 18, DNA near-tetraploid), showed a near-diploid tumour population with a DI between 0.90 and 1.07. In two of these tumours (No. 19 and No. 22), in addition to the DNA near-diploid cell population, a second cell fraction was observed with a near-tetraploid DNA index.

An example of complete allelic state of a PTC tumour (PTC-OV, case No. 24) is shown in Figure 2B. In the SNP-array analysis relative limited chromosomal alterations were identified in this case. In contrast to FTC-OV, the other histological variants showed relatively limited numbers of chromosomal aberrations. Allelic state [A], indicating physical loss, or [AA] (copy-neutral LOH) was observed for chromosome 22 in six out of eleven PTC or PTC-variant cases.

**Figure 4:**
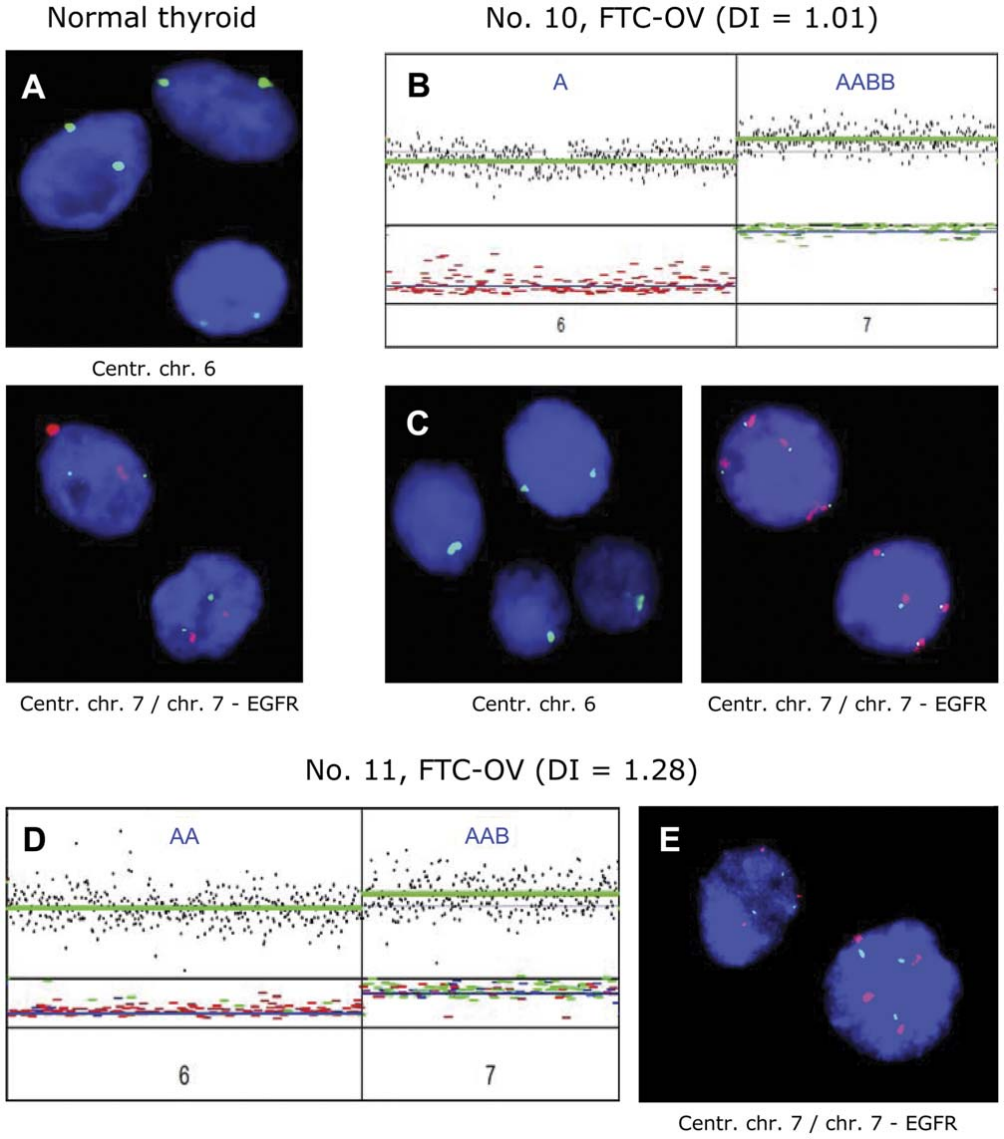
Interphase FISH analysis in relation to allelic state analysis. In FTC-OV, chromosome 6 was always observed in allelic state [A] or [AA], whereas chromosome 7 was always retained in a heterozygous state or amplified heterozygous state. To confirm these results, interphase FISH was performed for chromosomes 6 and 7. Examples are shown, see also Table 1. **A**. FISH on normal thyroid epithelium. **B** and **C** show FTC-OV case No. 10. **B**: Allelic state analysis illustrating allelic state [A] for chromosome 6 and allelic state [AABB] for chromosome 7. **C**. Left panel: green signal, centromere 6 shows 1 copy, confirming the allelic state [A] in three of the four nuclei. Right panel: four green and four red signals representing EGFR and centromere 7, respectively and confirming the [AABB] allelic state. **D–E** show FTC-OV, case no 11. **D**. The allelic state [AAB] of chromosome 7 could not be confirmed definitively and showed a mixture of nuclei containing three green (EGFR) and three red (centromere) signals or four green and four red signals, respectively (**E**). This may be due to intra-tumour heterogeneity.

FISH analysis: To confirm the copy number of chromosomes 6 and 7, FISH analysis was performed on interphase nuclei isolated from nine FTC-OV and three PTCs (Table 1, Figure 4). Centromeric probes for both chromosome 6 and 7 and an additional probe on 7p (*EGFR* locus) were used. The previously determined copy numbers were confirmed in the vast majority of cases, with 3 exceptions. Two of these exceptions involved chromosome 7 of tumours no 11 and 12. In tumour no 11, the LAIR results for chromosome 7 (Figure 4D) were interpreted as three chromosomal copies in an imbalanced state (allelic state [AAB]) while FISH (Figure 4E) clearly showed a mixture of three and four copies, both for the centromeric and the *EGFR* specific probes. In tumour no 12, the chromosome 7 discordance might be attributable to intra-tumour heterogeneity as this sample corresponds to a bimodal histogram where two cell populations are present.

### Validation of the near-homozygous phenotype found in recurrent FTC-OV

In order to validate our findings we additionally studied the frozen tissue of twenty thyroid tumours using high-density SNP-arrays. The validation cohort consisted of three follicular adenomas (FA, one partially oncocytic), five anaplastic thyroid carcinomas (ATC, one partially oncocytic), one FTC/PTC, one FTC, one FTC partially oncocytic, two FTC-OVs, two minimal invasive FTC-OVs, and five PTCs. All patients with ATC died within 5 months after treatment. Of the remaining cases five showed recurrences (see Table 2 and for detailed information Table S2).

Two FTC-OV (Table 2, No's 38 and 39) and one FTC, partially OV (No. 37) showed the characteristic near-homozygous phenotype as described above. From one FTC-OV patient (Table 2, No. 39) an FFPE sample was analyzed in the initial series (Table 1, No. 9). The frozen sample was comparable with the FFPE sample and showed chromosomes 1–11, 15–17 and 19–22 in identical heterozygous or homozygous states, respectively. Only chromosomes 12, 13 and 19 showed homozygous in the FFPE sample while these were retained in the frozen sample (compare Figure 3, No. 9 with Figure S2, No. 39).

The near-homozygous phenotype was not observed in any other case in the validation series. However one ATC with partially oncocytic features (Table 2 and Table S2, No. 35) showed allelic imbalances for all chromosomes except chromosome 7 (Figure S2).

### Mutation analysis of *EGFR*, *BRAF*, RAS genes and *PIK3CA*

A summary of the detected mutations in the initial cohort is presented in Table 1. Mutations in the RAS genes and *PIK3CA* were rarely found. One FTC-OV case (No. 11) and the PTC Tall Cell tumour (No. 27) showed a *PIK3CA* (c.86276A>G, p.H1047R) mutation. Two tumours (FTC No. 1 and PTC No. 22) showed *NRAS* (c.2987A>G, p.Q61R) mutations. No mutations were detected in *EGFR*. Of the thirteen PTCs and PTC variants analysed, ten (77%) showed the activating *BRAF* mutation, c.1799T>A, p.V600E. The *BRAF* mutation was not detected in the FTC-OV (n = 10) or FTC (n = 4) subgroups. In the validation cohort 4 *BRAF* c.1799T>A, p.V600E mutations were detected, all in PTC. No mutations were found in *KRAS* or *PIK3CA*.

## Discussion

We initially characterised recurrent NMTC by combining genome-wide SNP-array analysis with DNA flow cytometry. We clearly showed that recurrent oncocytic follicular thyroid carcinoma (FTC-OV) is characterised by genomic haploidisation, frequent endoreduplication of a DNA near-haploid genome with especially retention of a heterozygous chromosome 7 in all the cases. The latter suggests a strong association of genomic haploidisation with mitochondrial defects. In a subsequent validation cohort this genomic phenotype found in FTC-OV was also observed in two additional recurrent thyroid cancers with oncocytic features. There were also 4 cases showing morphologically partially or overt oncocytic features (one benign FA and one ATC and 2 non recurrent minimal invasive FTC-OV) that did not show the characteristic genomic phenotype described above. The one ATC (partially OV) however did show genome-wide imbalances with the exception of chromosome 7. Tumour cell percentage was high enough in this particular sample, so only tumour heterogeneity could be an explanation.

Our flow cytometric findings differ from previous single parameter flow cytometric DNA content measurements of FTC-OV carcinomas [23], [24]. In contrast, our multiparameter flow cytometric method for FFPE samples allows the use of stromal cells as a DNA diploid reference [20]. Previously, defining a reliable DNA content reference was problematic [25]. In the single-parameter approach, the left peak of a bimodal DNA histogram was assumed to represent the normal stromal cell fraction in all cases [26]. This approach can lead to the mistaken designation of a DNA near-haploid FTC-OV as DNA diploid. To date, all studies measuring DNA content of FTC-OV have defined these tumours as DNA diploid or aneuploid [23]. In our opinion the DNA histograms published in 1988 by McLeod et al. [24] show DNA near-haploidy in FTC-OV, but these authors did not elaborate on this observation (see Figure S1). Haploidisation has only been widely documented in chondrosarcomas [27] and is found sporadically in solid tumours. Even so, we found one report of a renal oncocytoma with a near-haploid karyotype [28]. One study on copy number alterations by SNP-arrays showed a near-homozygous like phenotype in one of the fifteen renal oncocytoma cases [29]. Others did not report these specific genomic alterations [30], [31]. Oncocytic thyroid tumours have been studied by CGH technology. A limitation is that with CGH possible regions of chromosomal homozygosity cannot be detected. Haploidisation is missed with CGH due to the fact that normalisation of CGH patterns is based on average genomic content. Interestingly, one well documented array-CGH study of 13 thyroid FTC-OV and 15 FA-OV by Wada et al. showed highly comparable copy number alterations [9]. Just like our investigation they found increased signal intensities for chromosomes 5, 7, 12, 17, 19 and 20. Relative losses were mostly restricted to chromosomes 2 and 9. Noteworthy, these authors (also) suggested an association between these specific numerical chromosomal aberrations and recurrence. Five Patients (n = 5) without chromosomal aberrations did not recur, whereas 5 of 8 patients with chromosomal aberrations showed a recurrence. In addition, XTC.UC1 cells [32], the only known model for oncocytic thyroid cancer [33], [34], shows a CGH profile [7] highly comparable with those found by Wada et al. [9].

**Figure 5:**
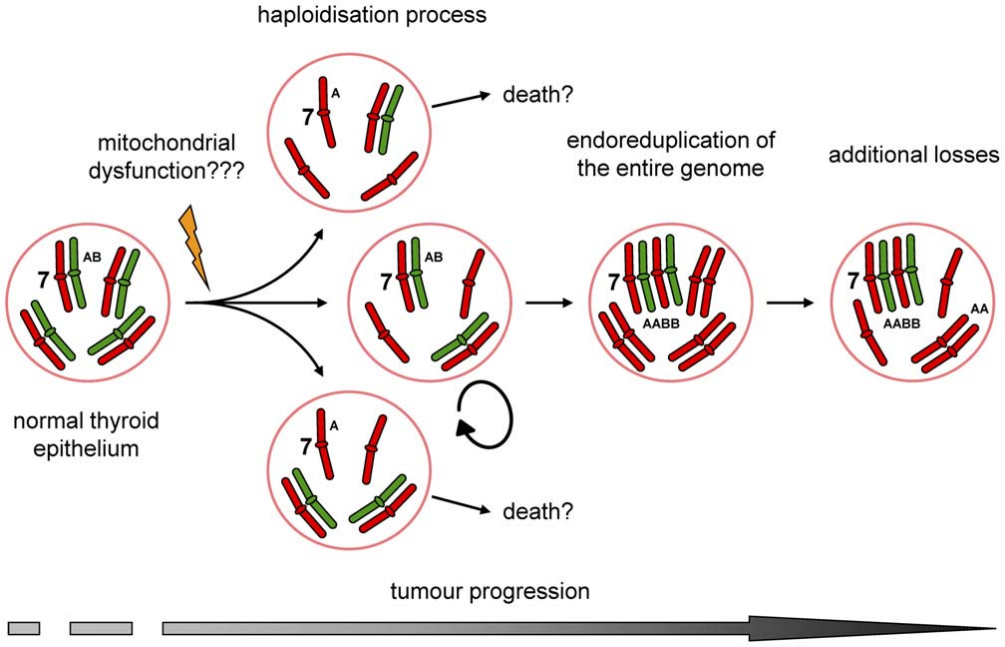
Hypothetic model of oncocytic follicular thyroid carcinoma development and progression. Mutations in mtDNA underlie low levels of ATP productions. In order to compensate for these low energy levels mitochondria carrying these mutations proliferate and accumulate in the cytoplasm of affected cells. Oncocytic FTC is also characterised by a mitochondria-rich cytoplasm, are known to harbour mtDNA mutations (mainly complex I) and do show a disturbed energy production. DNA replication and progression through the cell cycle are energy demanding processes. Low energy levels might also disturb normal formation and function of the mitotic spindle, resulting in an unbalanced mitosis. Cells that have lost chromosomes during several rounds of cell division become DNA near-haploid (haploidisation process). Sustaining a near-haploid genome may require less energy than of normal 2n cells. Consequently near-haploid cells show a growth advantage and are selected for during tumour development. Maintaining chromosome 7 in heterozygous state ([AB]) seems to be essential for tumour survival. This might be indicative for the presence of genes playing an important role in oncocytic FTC. Endoreduplication of these genes into an [AABB] allelic state increases the gene dosage which might be beneficial for further progression.

Our observations suggest a possible relation between the FTC-OV phenotype and haploidisation/endoreduplication. However, the underlying molecular mechanism remains to be elucidated. Possible mechanisms are abnormal mitosis [35] or meiosis-like events. Alternatively, the process of haploidisation may well be driven by a lack of cellular energy [36] which in FTC-OV may be caused by disruptive mutations in mitochondrial respiratory complex I [37]. Low mitochondrial ATP levels [38] may compel a decrease in DNA content in order to promote cell survival. The loss of entire chromosomes might be a step-wise process and might be associated with tumour progression (Figure 5). This is supported by our findings in the FFPE and frozen sample of the primary tumour of the same patient which differed in allelic state for chromosomes 12, 13 and 19 (Sample No. 9 and 39, respectively). However, the endoreduplication seen in several of the tumours also demands further explanation.

Taken together, these and our observations suggest a relation between mitochondrial complex I deficiencies, lack of energy and loss of entire chromosomes during tumour development and progression. Mitochondrial complex I deficiencies have also been found in other types of tumours [39], amongst chromophobe renal cell carcinoma [40] and renal oncocytoma [41]. Also chromophobe renal cell carcinoma shows frequent losses of entire chromosomes [29]–[31], [42] and as mentioned earlier renal oncocytoma does show a highly comparable genomic pattern in rare cases [29].

High frequencies of LOH in FTC-OV were previously observed using microsatellite analysis [43], [44]. The authors of one study actually suggested the involvement of chromosomal loss, but did not recognise possible DNA haploidisation since the FTC-OV tumours were assumed to be DNA diploid or DNA aneuploid [45]. Retention of chromosome 7 has been described previously, both for FTC-OV and FTC [45]. This might indicate that retention of the heterozygous state in this chromosome is important for tumour survival in recurrent FTC-OV. Interestingly, an association between accumulation of mitochondria in oncocytic lesions and trisomy of chromosome 7 has also been described [46]. Several important oncogenes are located on chromosome 7, including *cMET*, *BRAF* and *EGFR*. Three thyroid cancer cases with activating EGFR mutations have been described, two of which showed a favourable response when treated with EGFR tyrosine-kinase inhibitors [47], [48]. No activating mutations in *EGFR* were identified in our cohort and only two samples showed a mutation in an EGFR downstream signalling molecule (*NRAS* and *PIK3CA*).

Only a limited number of gross chromosomal aberrations were identified in FTC and PTCs. PTCs showed frequent LOH on chromosome 22q, in concordance with a previous study [49]. An array CGH study in PTCs showed higher percentages of chromosomal aberrations [6]. However, in contrast to our study with *BRAF* c.1799T>A, p.V600E in 77% of the PTCs, very low frequencies of this mutation were found in that particular study. These findings further support the concept of different routes of tumour development in PTC.

Also in concordance with previous studies, DNA ploidy measurements on PTCs showed mainly diploidy or near-diploid DNA content. The three PTC-OVs (carrying *BRAF* c.1799T>A) did not show the typical haploidisation/endoreduplication seen in FTC-OV but were comparable to non-oncocytic PTC. Thus, haploidisation might be frequent in oncocytic tumours, but it is unlikely that all subtypes of oncocytic tumours show haploidisation. It is known that the BRAF p.V600E mutant protein in PTC translocates to the outer membrane of mitochondria and seems to be involved in high glucose uptake rate and reduced mitochondrial oxidative phosphorylation and ATP synthesis [50]. To compensate for reduced ATP synthesis, the mitochondrial mass expands, resulting in the typical oncocytic phenotype. The mitochondrial proliferation found in PTC-OV might therefore be secondary to the *BRAF* c.1799T>A mutation. Still, although speculative, the effects on the cellular energy supply of mutations in nuclear encoded mitochondrial genes might be different from mutations in mitochondrial encoded genes.

In summary, we showed for the first time that FTC-OV is frequently characterised by initial genomic haploidisation, showing monosomies of entire chromosomes but with retention of chromosome 7 in a heterozygous state. Endoreduplication of the previous near-haploid genome was observed in half of the FTC-OV cases. Retention of chromosome 7 might be essential for tumour cell survival in recurrent FTC-OV and may open new avenues to targeted therapies. A cohort of 20 cases confirmed our findings. The near-homozygous phenotype is associated with FTC-OV and might reflect a more aggressive disease. Whether the oncocytic cell phenotype and mitochondrial dysfunction in FTC-OV are directly related to these chromosomal aberrations remains to be established.

## Supporting Information

Table S1 **Primers used for hot-spot mutation analysis of** ***EGFR*** **,** ***NRAS*** **,** ***HRAS*** **,** ***KRAS*** **,** ***BRAF*** **and** ***PIK3CA*** **in NMTC.**
(DOC)

Table S2 **Detailed overview of the validation cohort.**
(XLS)

Figure S1 **Examples of DNA content analysis of FFPE oncocytic follicular thyroid carcinomas by McLeod et al.** [**24**] **.**
(DOC)

Figure S2 **Summary of high-density genomic patterns found in the validation cohort (n = 20).**
(DOC)
