# Supplementary material for: Genome Haploidisation with Chromosome 7 Retention in Oncocytic Follicular Thyroid Carcinoma
Source: PLoS One. 2012 Jun 1;7(6):e38287. doi: 10.1371/journal.pone.0038287 (PMC3365880; doi:10.1371/journal.pone.0038287)
Supplement: Table S1 — Primers used for hot-spot mutation analysis of EGFR , NRAS , HRAS , KRAS , BRAF and PIK3CA in NMTC. (DOC) [file pone.0038287.s001.doc]

**Supplementary Table S1**

Primers used for hot-spot mutation analysis of *EGFR*, *NRAS*, *HRAS*, *KRAS*, *BRAF* and *PIK3CA* in NMTC

| **Gene** | **Exon** | **Primer sequence with M13 tails (5’→3’)** |
| --- | --- | --- |
| EGFR | 18 | TGTAAAACGACGGCCAGTTCCCCACCAGACCATGAGAG |
|  |  | CAGGAAACAGCTATGACCAGGGCTGAGGTGACCCTTGT |
| EGFR | 19 | TGTAAAACGACGGCCAGTGGCAGCATGTGGCACCATCTCAC |
|  |  | CAGGAAACAGCTATGACCAGCCATGGACCCCCACACAGC |
| EGFR | 20 | TGTAAAACGACGGCCAGTACTGACGTGCCTCTCCCTCCC |
|  |  | CAGGAAACAGCTATGACCCCTTATCTCCCCTCCCCGTATCTCC |
| EGFR | 21 | TGTAAAACGACGGCCAGTCTGTCCCTCACAGCAGGGTCTTC |
|  |  | CAGGAAACAGCTATGACCGGTGTCAGGAAAATGCTGGCTGACC |
| NRAS | 2 | TGTAAAACGACGGCCAGTggtttccaacaggttcttgc |
|  |  | CAGGAAACAGCTATGACCcactgggcctcacCTCTATG |
| NRAS | 3 | TGTAAAACGACGGCCAGTcacacccccagGATTCTTAC |
|  |  | CAGGAAACAGCTATGACCTGGCAAATACACAGAGGAAGC |
| HRAS | 2 | TGTAAAACGACGGCCAGTcaggagaccctgtaggaggac |
|  |  | CAGGAAACAGCTATGACCcacCTCTATAGTGGGGTCGTATTC |
| HRAS | 3 | TGTAAAACGACGGCCAGTgaggctggctgtgtgaactc |
|  |  | CAGGAAACAGCTATGACC TGATGGCAAACACACACAGG |
| KRAS | 2 | TGTAAAACGACGGCCAGTTCGACCCAGGATCCAACTTGCTGAAAATGACTGAATATAAACTTGT |
|  |  | CAGGAAACAGCTATGACCATCCAGTACTTGAGAATTCCATCTAGCTGTATCGTCAAGGCACTC |
| KRAS | 3 | TGTAAAACGACGGCCAGTccagactgtgtttctcccttc |
|  |  | CAGGAAACAGCTATGACCAAGAAAGCCCTCCCCAGTC |
| BRAF_V600E | 15 | TGTAAAACGACGGCCAGTTTCATGAAGACCTCACAGTAAAAATAGGT |
|  |  | CAGGAAACAGCTATGACCTGGGACCCACTCCATCGA |
| PIK3CA | 9 | TGTAAAACGACGGCCAGTGGGAAAATGACAAAGAACAGC |
|  |  | CAGGAAACAGCTATGACCTCCATTTTAGCACTTACCTGTGAC |
| PIK3CA | 20 | TGTAAAACGACGGCCAGTCTGAGCAAGAGGCTTTGGAG |
|  |  | CAGGAAACAGCTATGACCcctatgcaatcggtctttgc |
