# Supplementary material for: Genome Haploidisation with Chromosome 7 Retention in Oncocytic Follicular Thyroid Carcinoma
Source: PLoS One. 2012 Jun 1;7(6):e38287. doi: 10.1371/journal.pone.0038287 (PMC3365880; doi:10.1371/journal.pone.0038287)
Supplement: Figure S1 — Examples of DNA content analysis of FFPE oncocytic follicular thyroid carcinomas by McLeod et al. [24] . (DOC) [file pone.0038287.s003.doc]

**Supplementary Figure S1**

Examples of DNA content analysis of FFPE oncocytic follicular thyroid carcinomas by McLeod et al.


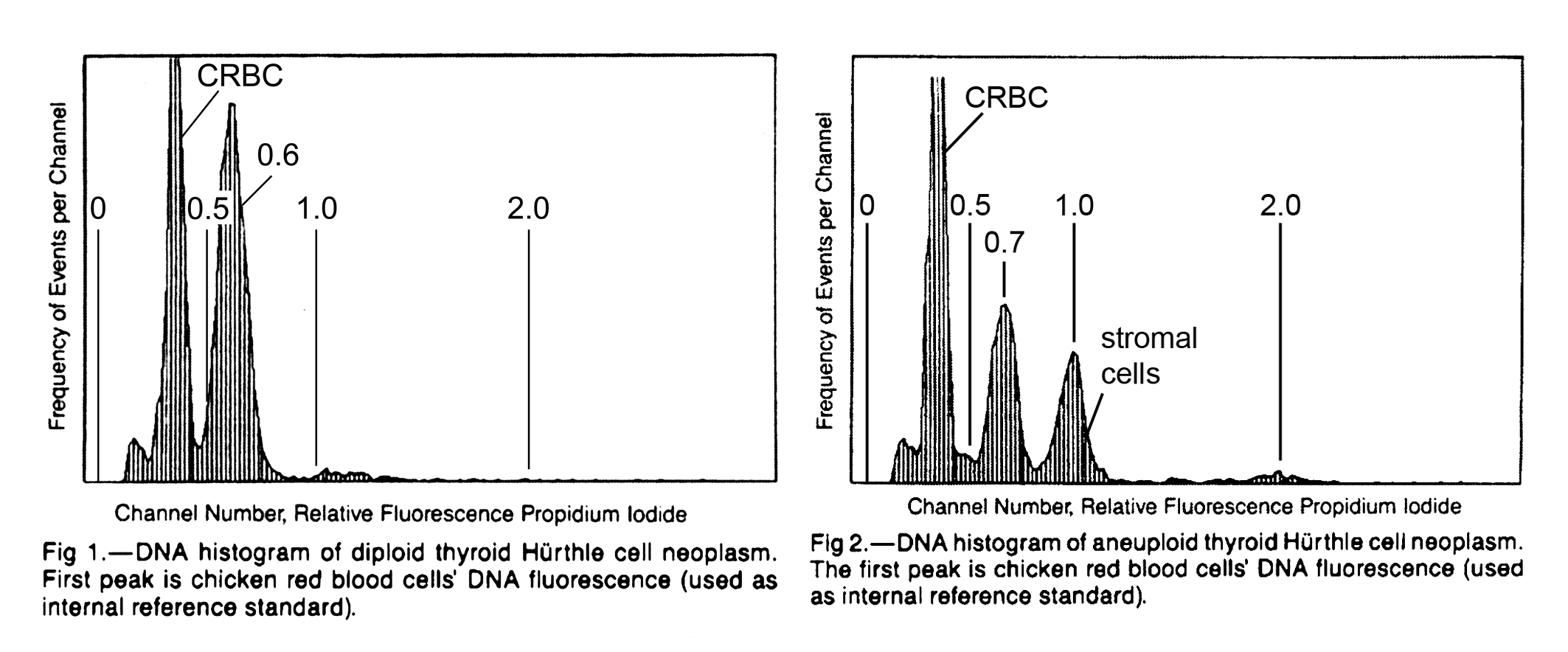


Copies were made of Figures 1 and 2 from the study by McLeod et al., Arch Surg 1988;123:849-54 and an arbitrary linear scale was added with markers at positions 0, 0.5, 1 and 2.

According to McLeod et al. the first peak of each histogram (see Figures 1 and 2 above, with adaptations from Corver et al.) represents propidium iodide stained chicken red blood cells (CRBC). However, the DNA content of CRBC is 35% of that of nucleated normal human cells. Although FFPE samples were used, which are known to harbour fixation artefacts affecting a stoichiometric DNA labelling, the CRBC are still positioned at approximately one third of the arbitrary linear scale compared to the position of the peak at 1.0 in Figure 2. Thus the peak at position 1.0 represents G_0_G_1_ DNA diploid stromal cells and the peak right of the bar at 0.5 must be near-haploid DNA with an estimated DNA index of 0.7. The peak at 2.0 is composed of nuclei in the G_2_M phase of the cell cycle of the DNA diploid population.

Due to the sometimes low percentages of normal stromal cells that are present in oncocytic follicular thyroid carcinomas, only one cycling population (excluding the peak from CRBC) can be resolved by single parameter DNA flow cytometry, and DNA histograms can be misinterpreted (Figure 1). Using the same arbitrary scale for Figure 1 as for Figure 2, it can now be seen that the second peak must represent DNA near-haploid tumour cells with an estimated DNA index of 0.6. These cells are clearly not DNA diploid.
