# Supplementary material for: Genome Haploidisation with Chromosome 7 Retention in Oncocytic Follicular Thyroid Carcinoma
Source: PLoS One. 2012 Jun 1;7(6):e38287. doi: 10.1371/journal.pone.0038287 (PMC3365880; doi:10.1371/journal.pone.0038287)
Supplement: Figure S2 — Summary of high-density genomic patterns found in the validation cohort (n = 20). (DOC) [file pone.0038287.s004.doc]

**Supplementary Figure S2**

Summary of high-density genomic patterns found in the validation cohort (n = 20). Data of chromosomes 1 – 22 are shown.


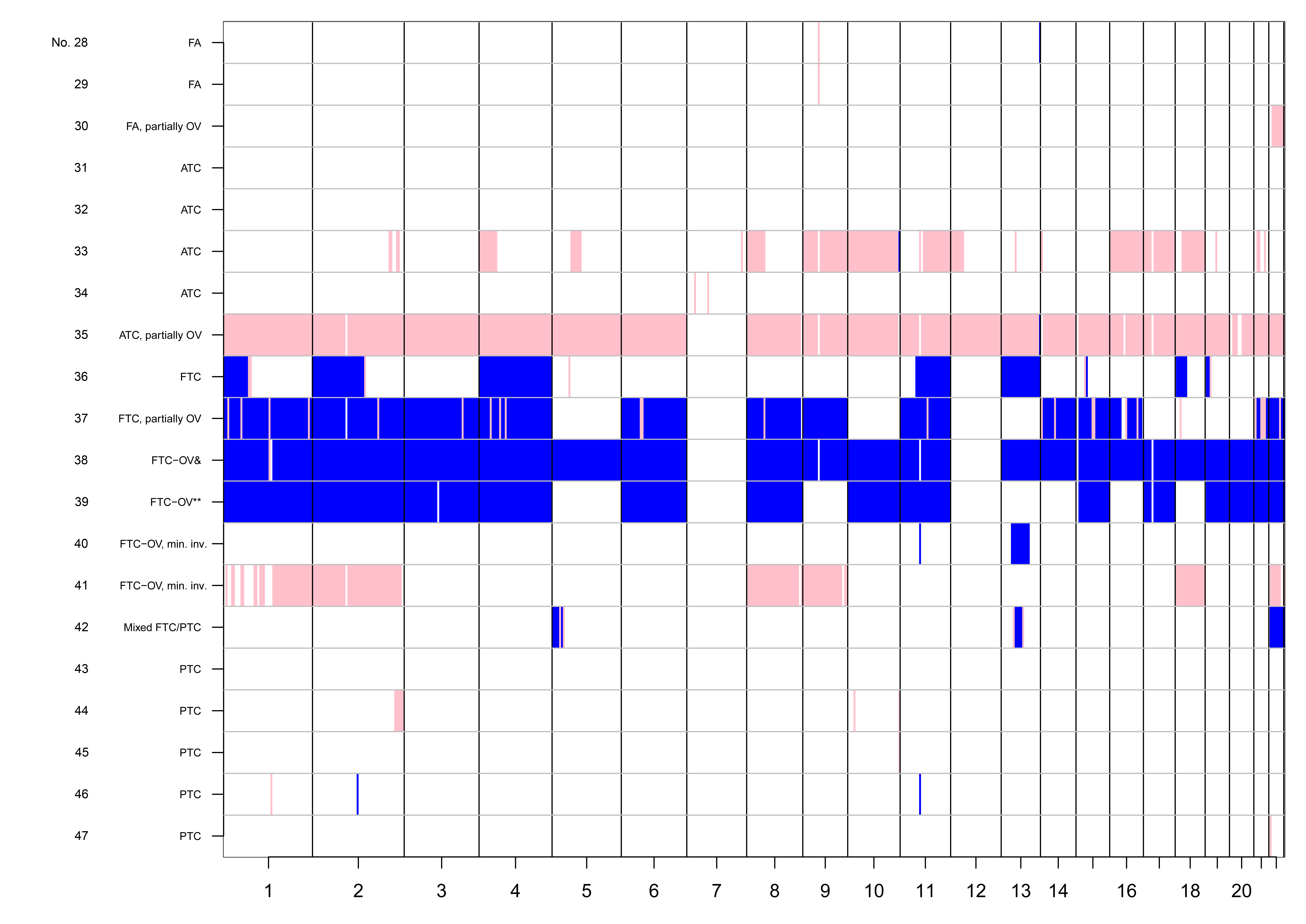


White = retention

Pink = allelic imbalance

Blue = loss of heterozygosity

^&^ = other tumour fraction dedifferentiated to ATC

** = sorafenib study, FFPE sample No. 9
